# Smart Enough? What Italian Farmers Reveal About Dairy Cow Technologies: A Survey Study

**DOI:** 10.3390/ani16081170

**Published:** 2026-04-11

**Authors:** Martina Lamanna, Edlira Muca, Chiara Montano, Marco Bovo, Francesco Petretto, Riccardo Colleluori, Andrea Formigoni, Damiano Cavallini

**Affiliations:** 1 Department of Veterinary Medical Sciences, Alma Mater Studiorum—University of Bologna, 40064 Ozzano dell’Emilia, Italy; chiara.montano2@unibo.it (C.M.); riccardo.colleluori2@unibo.it (R.C.); andrea.formigoni@unibo.it (A.F.); damiano.cavallini@unibo.it (D.C.); 2 College of Veterinary Medicine, University of Al Dhaid, Sharjah P.O. Box 27272, United Arab Emirates; e.muca@uodh.ac.ae; 3 Department of Agricultural and Food Sciences, University of Bologna, 40126 Bologna, Italy; marco.bovo@unibo.it; 4 Independent Researcher, 07100 Sassari, Italy; petretto.francesco@gmail.com

**Keywords:** artificial intelligence, dairy cow, farmer perception, Precision Livestock Farming, user-centred design

## Abstract

Precision Livestock Farming (PLF) tools are increasingly used in dairy production, but their success depends on farmers’ perceptions, needs and investment capacity. This study explores the current use of digital technologies, satisfaction levels and future expectations among Italian dairy farmers. An online questionnaire with 19 questions collected 53 complete responses between May and November 2025. Most of the farms were free-stall Holstein dairy farms located in the Po Valley and managed by relatively young and well-educated farmers, many of whom had a background in animal production. The adoption of PLF tools was widespread: management software (73.6%), automated total mixed ration (TMR) preparation (66.0%), heat stress mitigation systems (62.3%) and collar sensors (52.8%) were the most adopted technologies. Satisfaction with current tools was high, although installation costs and poor system integration were consistently identified as major constraints. Farmers expressed clear priorities for future devices, particularly early diagnosis of health problems, calving, heat, lameness, and feeding and rumination functions. The results suggest that PLF in Italian dairy systems is moving from the adoption phase to that of consolidation. However, improvements in interoperability, affordability and farmer-centred design remain essential to support a wider and more equitable spread of the technology across the sector.

## Simple Summary

This study used an online questionnaire to understand how Italian farmers use and perceive Precision Livestock Farming technologies. The responses showed that many farms are already highly digitised and rely on management software, automated feeding systems and wearable sensors. Farmers generally reported positive experiences with these tools but consistently highlighted two main challenges: high installation costs and poor compatibility between systems from different manufacturers. When asked what features future devices should have, farmers prioritised early diagnosis of health issues, calving, heat and lameness, functions that play a key role in daily herd management and animal welfare. The strong correlation found between past and planned investments suggests that farms already engaged in digitalisation are the most likely to continue investing, potentially widening the technology gap within the sector. Overall, the survey highlights the need for more accessible, interoperable Precision Livestock Farming solutions designed around the practical needs of farmers in order to support greater and more effective adoption across Italian dairy production.

## 1. Introduction

Over the past two decades, PLF has progressively transformed the way livestock systems are conceived, monitored, and managed [1]. PLF integrates engineering, animal science and data analytics to achieve continuous, automated control of animals and their environment through sensors, predictive algorithms and digital platforms capable of processing real-time information on health, behaviour, production and welfare. These tools support timely interventions to improve productive and reproductive performance [2], prevent diseases [3], optimise resource use and reduce environmental impact.

The increasing size of dairy herds and the reduced time available per animal make continuous human supervision challenging, reinforcing the role of automated systems as both technological and managerial aids [4]. PLF does not replace farmers’ experience but enhances it by making the assessment of animal condition more objective and enabling proactive management [5]. Current applications include behavioural monitoring, such as feeding and rumination [6], early detection of metabolic and reproductive disorders, and environmental and welfare monitoring through diverse sensor technologies [7]. Data processed through machine-learning algorithms can generate early alerts and integrate with farm management systems to support faster, evidence-based decisions [8].

Beyond technological innovation, PLF represents a conceptual shift from empirical observation to continuous measurement and prediction. Animals are increasingly viewed as individual biological systems whose dynamic responses can be monitored to detect early alterations in health and welfare [9]. This individual-based approach aligns with broader sustainability goals: improved nutritional precision, reduced waste, enhanced feed efficiency and associated reductions in greenhouse gas emissions per unit of product [10,11]. In parallel, PLF contributes to transparency and traceability in a public debate where the environmental role of livestock is often misrepresented [12,13,14].

Despite these advantages, PLF adoption in Italy remains heterogeneous. Barriers include high investment costs, lack of interoperability, maintenance challenges and varying levels of trust in digital tools [15]. Moreover, sensor accuracy alone does not guarantee effectiveness; farmers’ responses to alerts and their ability to integrate digital information into daily routines are fundamental, and excessive notifications may lead to alert fatigue. Understanding farmers’ perceptions and needs is therefore essential for designing solutions that are both technically valid and practically adoptable [16].

The present study aims to provide an updated assessment of how Italian dairy farmers adopt, use and evaluate PLF technologies. Its specific objectives are as follows:

- Describe the current diffusion of digital tools across different farm structures and geographical areas.
- Assess farmers’ satisfaction with existing PLF systems and identify the main technical and economic limitations perceived in practice.
- Quantify past and planned investments in PLF technologies and examine how these vary according to herd size, farmer age and geographical macro-area.
- Identify the monitoring functions farmers consider priorities for next-generation devices.

Previous surveys on PLF adoption in Italy have provided valuable but geographically limited insights. Abeni et al. [17] focused on a highly intensive dairy district in northern Italy (Cremona, Lombardy), highlighting a strong relationship between herd size and technology uptake. Similarly, Bianchi et al. [18] primarily analysed farms located in Lombardy, reporting high levels of technological adoption in intensive systems, but with limited representation from central and southern regions. In contrast, the present study provides a broader national perspective by including farms from multiple geographical macro-areas and production systems, and extends previous work by incorporating economic analyses of past and planned investments as well as farmer-defined priorities for future PLF development.

## 2. Materials and Methods

### 2.1. Ethical Considerations

This study was conducted in accordance with the ethical principles of the Regulation (EU) 2016/679 (General Data Protection Regulation, GDPR), and the University of Bologna guidelines for the protection of personal data in online surveys. In accordance with institutional guidelines for minimal-risk, non-interventional research involving adult participants and non-sensitive data, formal ethical approval was not required. The research was limited exclusively to the collection of opinions and perceptions through a voluntary questionnaire. Participation was voluntary and based on informed consent, which was obtained from all respondents prior to accessing the questionnaire

At the beginning of the questionnaire, participants were shown a brief introductory text outlining the purpose of the study, namely to support the design of multisensor PLF devices that are better aligned with farmers’ real needs. The introduction also indicated the estimated completion time (approximately 10 min) and clarified that all responses would be treated anonymously, unless participants explicitly chose to disclose their identity. All data were anonymised prior to analysis, and any contact information provided by participants was excluded from the analytical dataset. At the end of the survey, prior to submission, participants were shown the full privacy notice in compliance with Article 13 of the GDPR. This policy clarified that the data would be used exclusively for research, in accordance with the principles of lawfulness, fairness and transparency, and would be stored only for as long as necessary to achieve the stated objectives. The fields relating to contact information (farm name, email address and/or telephone number) were included in the final section of the questionnaire. These non-anonymous data were collected with the other responses in the Google Forms export file but were not disseminated, shared, or communicated to third parties, nor used for commercial purposes. The data has been collected exclusively for research management purposes, as indicated in the privacy policy. All results are presented in aggregated form, ensuring that individual respondents cannot be identified.

### 2.2. Study Design

This study was conducted as a cross-sectional online survey targeting Italian dairy farmers and farm operators. The aim was to gather updated information on the adoption, use, and perceived usefulness of PLF technologies, as well as farmers’ expectations for next-generation monitoring systems. Data were collected between May and November 2025.

The survey was created using the Google Forms web platform (Google LLC, Mountain View, CA, USA; accessed on 13 March 2025), chosen for its accessibility, mobile compatibility, and secure data handling. The questionnaire was designed by the research team of the Department of Veterinary Medical Sciences, University of Bologna, in November 2024. All items were formulated using clear and practical language to ensure usability for respondents with diverse educational levels and technical background. Before dissemination, the draft questionnaire was reviewed by industry professionals and informally tested across different digital devices to verify both comprehension and technical functionality. Participation was voluntary and uncompensated. At the beginning of the survey, respondents were presented with an introductory text explaining the purpose of the research, namely, to support the development of multisensor PLF devices better aligned with farmers’ operational needs.

To reach a broad audience of dairy producers across Italy, the questionnaire link and QR code were promoted via three channels:

- The Instagram page Stalla Didattica UNIBO, https://www.instagram.com/stalladidattica_unibo?igsh=MWg0d3A2anBiMXo3bQ== (accessed on 2 May 2025);
- An online news article published by the national livestock magazine Informatore Zootecnico (Edagricole, Piazza Galileo Galilei, 6, Bologna, Italy);
- The Instagram profile Il Piatto Consapevole, dedicated to agri-food communication and sustainability, https://www.instagram.com/ilpiattoconsapevole?igsh=NXR2bDFlY2hvZDk (accessed on 2 May 2025).

Incomplete submissions were excluded from the analysis. Prior to statistical analysis, the dataset underwent a structured screening process to ensure the quality, consistency, and reliability of the responses. Questionnaires were evaluated based on predefined inclusion and exclusion criteria, and responses were excluded if they were incomplete (i.e., missing data in key sections relevant to the study objectives), inconsistent or contradictory based on logical cross-checking of related items, or indicative of low-quality participation, such as unrealistically short completion times or uniform response patterns suggesting non-engaged answering. The final dataset was manually checked to identify potential duplicates, but none were detected. Following this data cleaning procedure, the final analytical sample consisted of *n* = 53 valid questionnaires, which were included in all subsequent analyses. All analyses were conducted using complete-case data, and no imputation procedures were applied.

### 2.3. Questionnaire Development and Structure

The final questionnaire consisted of 19 items, organised in three thematic areas followed by an optional contact section. A complete version of the instrument, including all questions and response options, is provided in Supplementary Material Section S1.

The first part collected background information on the respondent and farm characteristics, including province and production area (plain, hill, mountain), farm size (<10; 10–30; 30–50; 50–100; >100 hectares), number of lactating cows (<30; 31–60; 61–100; 101–200; >200), housing system (tie-stall, free-stall, pasture-based, mixed; the category “mixed” indicates farms that alternate between intensive and extensive management systems according to seasonal or operational needs throughout the year), and breeds reared (Holstein, Brown Swiss, Jersey, Simmental, or other). Additional items addressed the respondent’s role within the farm (owner, herd manager, employee), age class, education level, and whether their educational background was relevant to their current professional activity.

The second part explored the degree of digitalisation and farmers’ experience with PLF technologies. Respondents could select all tools currently used on the farm from a comprehensive list including software for herd management, robotic or automated milking systems, wearable sensors (collars, pedometers, ear-tags, tail sensors), systems for body weight and body condition score assessment, environmental monitoring sensors, heat-stress mitigation systems, feed-pushing devices, robotic or automated feed preparation systems, integrated mixer wagon technologies, Near Infrared Spectroscopy (NIRS) systems for feed analysis, water-intake monitoring systems, manure-scraping robots, and video surveillance systems. A “no technology” option was also available. For the purpose of this study, a distinction was made between advanced Precision Livestock Farming (PLF) technologies and general farm mechanisation. In particular, systems related to feed preparation (e.g., TMR preparation or mixer wagon technologies) were considered PLF-related only when associated with automated or sensor-based functionalities capable of recording, adjusting, or optimising feeding parameters, rather than conventional mechanical equipment without digital integration. Similarly, heat-stress mitigation systems included both basic mechanical solutions (e.g., fans or sprinklers) and more advanced systems incorporating environmental sensors or automated climate control. This distinction was considered during data interpretation to avoid overestimating the level of digitalisation. This section also included items assessing satisfaction with current technologies (five-point scale), perceived limitations (e.g., high costs, maintenance requirements, usability issues, difficulties in data interpretation, low precision, lack of system integration), and technological investments already made in the past five years or planned for the next five. The final thematic block focused on future needs and expectations. Respondents indicated up to three monitoring priorities should they invest in a new device such as feeding behaviour, rumination, health indicators, heat detection, heat stress, calving, lameness, or other parameters. Several items, particularly those concerning technologies used, perceived limitations, and desired monitoring functions, allowed multiple selections, meaning that the number of responses for these questions could exceed the total number of participants.

In the concluding contact section, participants were asked whether they had previously collaborated with universities or private companies and were invited to provide the farm name and an email or telephone number. As specified in the GDPR information notice, any personal data were stored exclusively for potential follow-up related to the research and were not used or shared for commercial purposes.

### 2.4. Data Management and Statistical Analysis

Data were exported from Google Forms web platform (Google LLC, Mountain View, CA, USA; accessed on 13 March 2025) in .xlsx format and processed using Microsoft Excel 2019 and JMP Pro 18 (SAS Institute Inc., Cary, NC, USA). Descriptive analyses were performed for all variables except for those related to investments. Categorical variables were summarised as absolute and relative frequencies and visualised through pivot tables, bar charts, and donut plots to describe the distribution of responses across categories.

For multiple-choice questions, such as those regarding technologies currently used, perceived limitations of PLF systems, and desired monitoring functions, each option was treated as an independent item. Therefore, the number of selections per option was counted rather than treating each questionnaire as a single response unit.

Analyses of investments were restricted to respondents who provided valid numeric values for the respective items; missing or non-quantifiable responses were excluded listwise. Inferential analyses were conducted to explore relationships between herd structure, farmer characteristics, and investment behaviour. Comparisons of past and planned investments between farms with ≤60 and ≥61 lactating cows were performed using the Mann–Whitney *U* test, due to the non-normal distribution of investment data. The same non-parametric test was applied to compare investment levels across geographical macro-areas (North vs. Centre, South, Islands), assessing whether regional context influenced willingness to invest.

To evaluate whether past investments were associated with future investment intentions, two complementary approaches were used:

- Pearson’s correlation coefficient r to quantify the strength of the linear association between the two continuous variables;
- Simple linear regression analysis to determine whether past investment statistically predicted future planned investment and to estimate the regression equation describing this relationship.

Finally, correlation analyses were performed to examine whether herd size (number of lactating cows) was associated with either past or planned investments, while investment patterns across age classes were explored descriptively. For the latter, no inferential tests were applied; instead, past and planned investments were summarised within each age group using the median and the 25th and 75th percentiles.

## 3. Results

A total of 53 complete questionnaires were collected for the final analysis. The dataset includes both single-choice items (e.g., farm size, number of lactating cows, age class) and multi-choice items (e.g., technologies used, perceived limitations, desired monitoring functions), providing a comprehensive overview of the structural, technological and managerial characteristics of the surveyed farms.

### 3.1. Structural and Demographic Characteristics of Farms and Respondents

**Figure 1:**
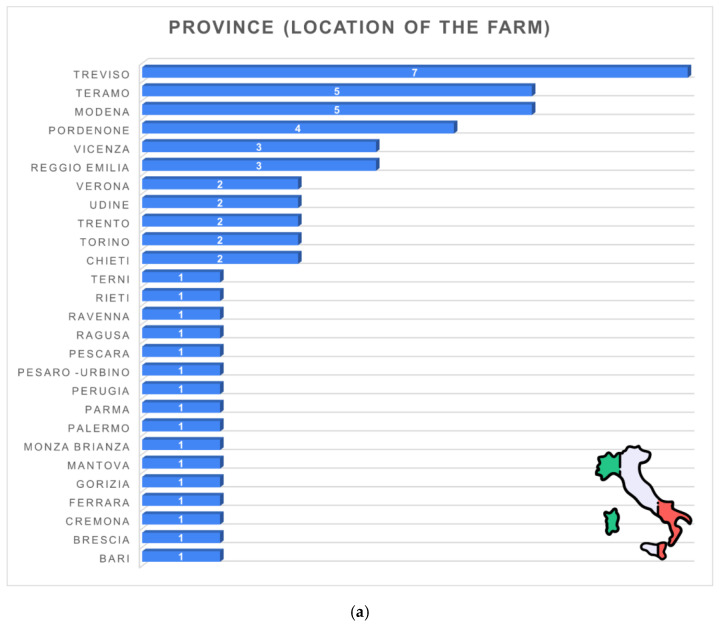

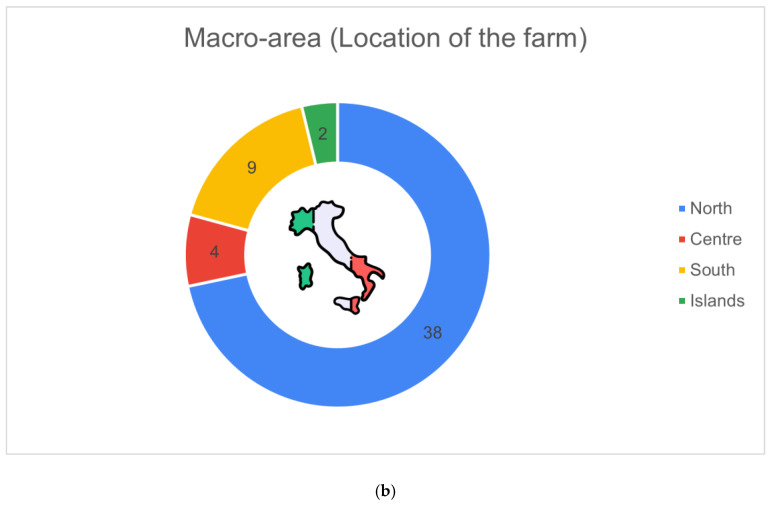
(**a**). Provincial distribution of the surveyed dairy farms (*n* = 53): Treviso (*n* = 7, 13.2%), Teramo (*n* = 5, 9.4%), Modena (*n* = 5, 9.4%), Pordenone (*n* = 4, 7.5%), Vicenza (*n* = 3, 5.7%), Reggio Emilia (*n* = 3, 5.7%), Verona/Udine/Trento/Torino/Chieti (each *n* = 2, 3.8%); other provinces (*n* = 1 each, 1.9%). (**b**). Distribution of surveyed dairy farms across Italian geographical macro-areas (*n* = 53): North (*n* = 38, 71.7%), South (*n* = 9, 17.0%), Centre (*n* = 4, 7.5%), Islands (*n* = 2, 3.8%).

Figure 1a illustrates the geographical distribution of the 53 participating dairy farms across Italy. The responses covered a wide range of provinces, although most farms were concentrated in the northern regions. The highest representation was from Treviso (*n* = 7), followed by Teramo (*n* = 5), Modena (*n* = 5), and Pordenone (*n* = 4). Additional clusters were observed in Vicenza and Reggio Emilia (each *n* = 3), while several provinces, including Verona, Udine, Trento, Torino, and Chieti, each accounted for two respondents. Many provinces were represented by a single farm (*n* = 1).

Figure 1b summarises the distribution of the 53 participating farms across the four main geographical macro-areas of Italy (North, Centre, South, Islands). Most respondents were located in the North (*n* = 38, 71.7%), confirming the strong concentration of dairy production in this area. A smaller proportion of farms was situated in the South (*n* = 9, 17.0%), followed by the Centre (*n* = 4, 7.5%). Only two farms were located in the Islands (Sardinia and Sicily), indicating limited representation from these regions (3.8%).

**Figure 2:**
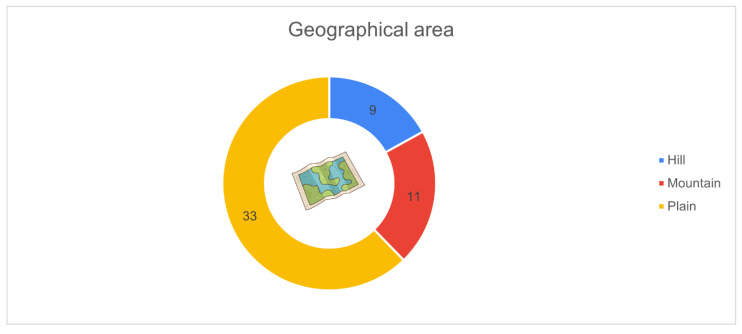
Distribution of farms surveyed by geographical production area (*n* = 53): plains (*n* = 33, 62.3%), mountains (*n* = 11, 20.7%), hills (*n* = 9, 17.0%).

Figure 2 shows the geographical distribution of the participating farms by production area. Most farms were in the plain regions (*n* = 33), representing 62.3% of the total respondents. A smaller proportion of farms were situated in hilly areas (*n* = 9, 17.0%) and mountain areas (*n* = 11, 20.7%).

**Figure 3:**
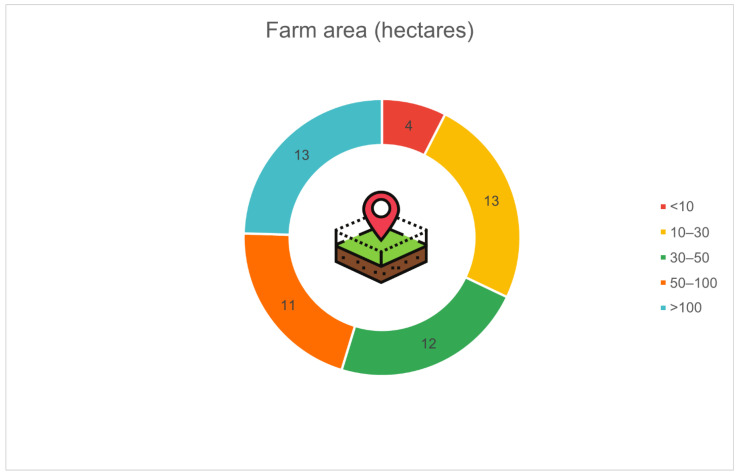
Distribution of surveyed farms by total farm area (hectares; *n* = 53): 10–30 ha (*n* = 13, 24.5%), >100 ha (*n* = 13, 24.5%), 30–50 ha (*n* = 12, 22.6%), 50–100 ha (*n* = 11, 20.8%), <10 ha (*n* = 4, 7.5%).

Figure 3 presents the distribution of farms according to their total surface area. The largest groups were farms with 10–30 hectares (*n* = 13, 24.5%) and over 100 hectares (*n* = 13, 24.5%), followed closely by farms of 30–50 hectares (*n* = 12, 22.6%) and 50–100 hectares (*n* = 11, 20.8%). Only a small proportion of farms reported an area of less than 10 hectares (*n* = 4, 7.5%).

**Figure 4:**
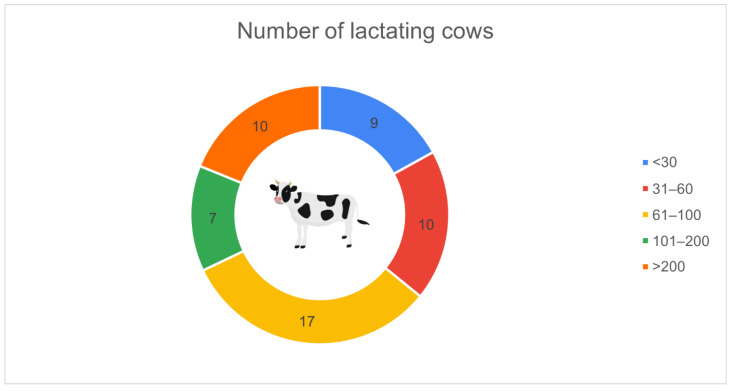
Distribution of surveyed farms by number of lactating cows (*n* = 53): 61–100 (*n* = 17, 32.1%), >200 (*n* = 10, 18.9%), 31–60 (*n* = 10, 18.9%), <30 (*n* = 9, 17.0%), 101–200 (*n* = 7, 13.2%).

Figure 4 illustrates the distribution of farms according to the number of lactating cows. The largest group consisted of farms with 61–100 cows (*n* = 17, 32.1%), followed by those with more than 200 cows (*n* = 10, 18.9%) and 31–60 cows (*n* = 10, 18.9%). Farms with fewer than 30 cows accounted for 9 respondents (17.0%), while 7 farms (13.2%) managed between 101 and 200 cows.

**Figure 5:**
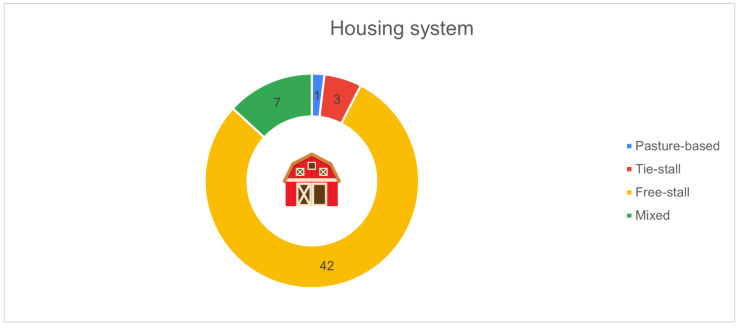
Distribution of surveyed farms by housing system (*n* = 53): free-stall (*n* = 42, 79.2%), mixed (*n* = 7, 13.2%), tie-stall (*n* = 3, 5.7%), pasture-based (*n* = 1, 1.9%).

Figure 5 shows the distribution of farms according to the housing system adopted. The free-stall system was by far the most common, reported by 42 farms (79.2%). A smaller proportion of respondents indicated the use of mixed systems combining different housing types (*n* = 7, 13.2%). Only three farms (5.7%) used tie-stall housing, and a single respondent (1.9%) reported a pasture-based system.

**Figure 6:**
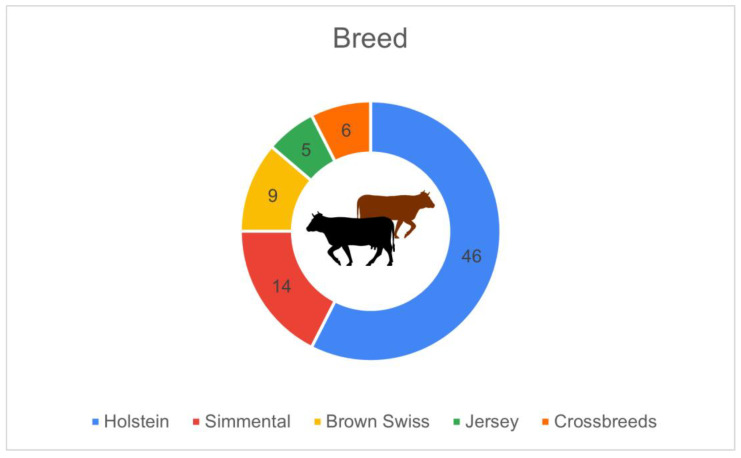
Breed composition of the dairy herds in the surveyed farms (*n* = 53): Holstein (*n* = 46, 86.8%), Simmental (*n* = 14, 26.4%), Brown Swiss (*n* = 9, 17.0%), crossbreeds (*n* = 6, 11.3%), Jersey (*n* = 5, 9.4%).

Figure 6 presents the distribution of dairy cow breeds across the surveyed farms. The Holstein breed was predominant, reported by 46 farms (86.8%), confirming its position as the leading dairy breed in Italy. The Simmental (Red and white) was present in 14 farms (26.4%), followed by Brown Swiss in 9 farms (17.0%), crossbreeds in 6 farms (11.3%), and Jersey in 5 farms (9.4%).

**Figure 7:**
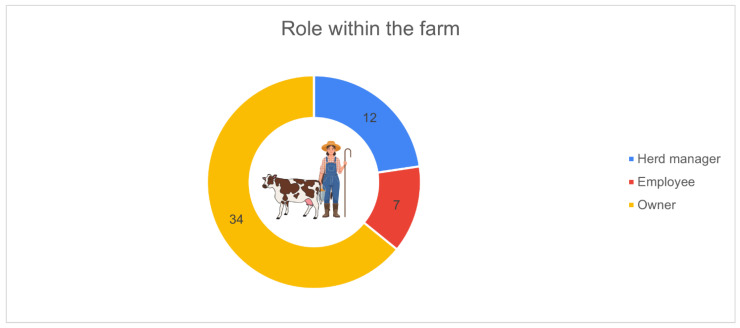
Role of the respondent within the farm (*n* = 53): owner (*n* = 34, 64.2%), herd manager (*n* = 12, 22.6%), employee (*n* = 7, 13.2%).

Figure 7 shows the distribution of respondents according to their role within the farm. The majority identified as owners (*n* = 34, 64.2%), followed by herd managers (*n* = 12, 22.6%) and employees (*n* = 7, 13.2%).

**Figure 8:**
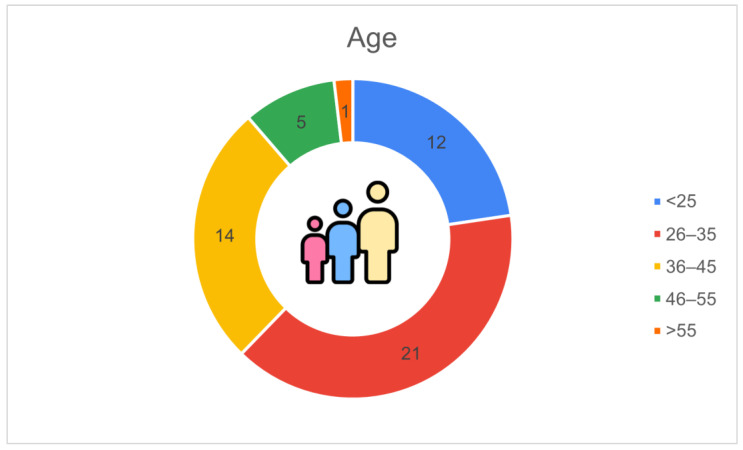
Age distribution of respondents (*n* = 53): 26–35 years (*n* = 21, 39.6%), 36–45 (*n* = 14, 26.4%), <25 (*n* = 12, 22.6%), 46–55 (*n* = 5, 9.4%), >55 (*n* = 1, 1.9%).

Figure 8 illustrates the age distribution of the respondents. The most represented group was 26–35 years old (*n* = 21, 39.6%), followed by 36–45 years (*n* = 14, 26.4%) and under 25 years (*n* = 12, 22.6%). The 46–55 age group accounted for 5 respondents (9.4%), while only one participant (1.9%) was over 55 years old.

**Figure 9:**
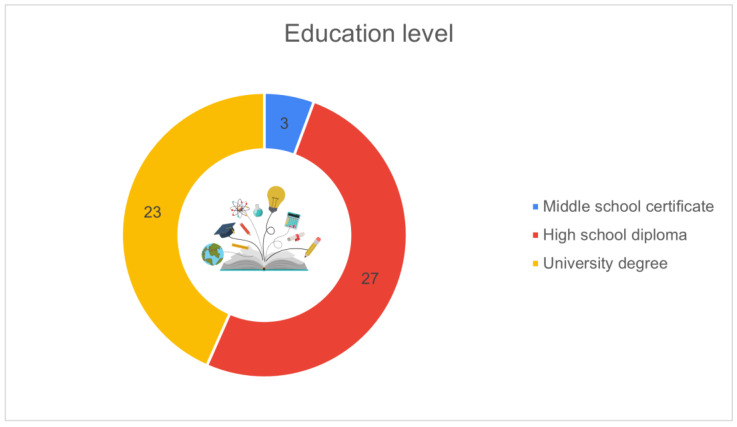
Education level of respondents (*n* = 53): high school diploma (*n* = 27, 50.9%), university degree (*n* = 23, 43.4%), middle school (*n* = 3, 5.7%).

Figure 9 presents the distribution of respondents according to their education level. Over half of the participants held a high school diploma (*n* = 27, 50.9%), while 23 respondents (43.4%) reported having a university degree. Only 3 respondents (5.7%) indicated middle school as their highest level of education.

**Figure 10:**
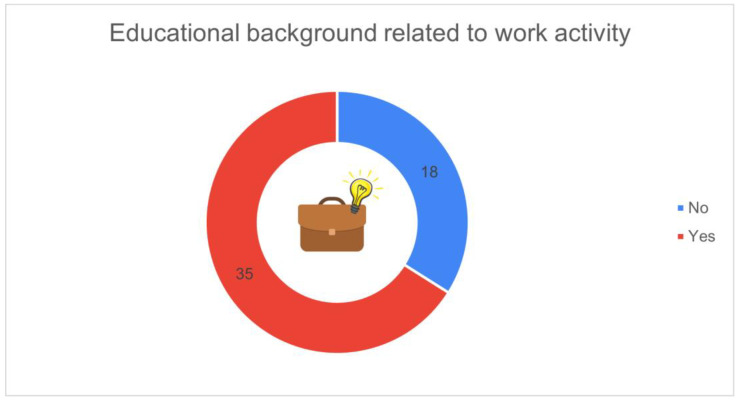
Educational background related to work activity (*n* = 53): yes (*n* = 35, 66.0%), no (*n* = 18, 34.0%).

Figure 10 shows whether respondents’ educational background was related to their current work activity. Most of the participants (*n* = 35, 66.0%) indicated that their studies were related to the agricultural or livestock sector, while 18 respondents (34.0%) reported a non-related educational background.

### 3.2. Technologies Currently Used

**Figure 11:**
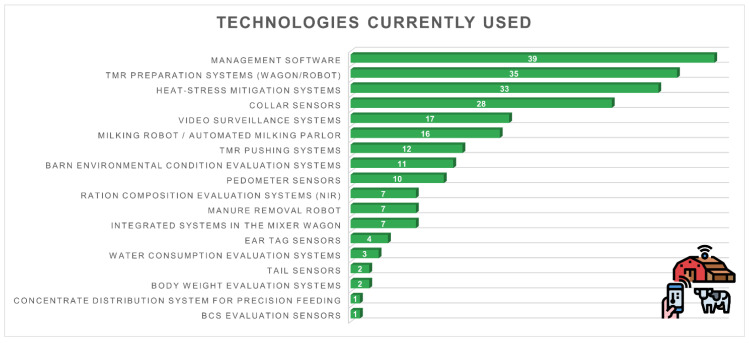
Technologies currently used on the farm (*n* = 53): management software (*n* = 39, 73.6%), TMR systems (*n* = 35, 66.0%), heat-stress mitigation (*n* = 33, 62.3%), collar sensors (*n* = 28, 52.8%), video surveillance (*n* = 17, 32.1%), milking robots/automated parlours (*n* = 16, 30.2%), TMR pushing systems (*n* = 12, 22.6%), environmental monitoring (*n* = 11, 20.8%), pedometers (*n* = 10, 18.9%), NIR systems (*n* = 7, 13.2%), manure robots (*n* = 7, 13.2%), integrated mixer systems (*n* = 7, 13.2%), ear-tag sensors (*n* = 4, 7.5%), water intake monitoring (*n* = 3, 5.7%), tail sensors (*n* = 2, 3.8%), body weight systems (*n* = 2, 3.8%), concentrate feeding systems (*n* = 1, 1.9%), BCS sensors (*n* = 1, 1.9%).

Figure 11 illustrates the technologies currently in use among the surveyed farms. The most widely adopted tools were management software (*n* = 39, 73.6%), followed by TMR preparation systems (wagon or robot, *n* = 35, 66.0%), heat-stress mitigation systems (*n* = 33, 62.3%), and collar sensors (*n* = 28, 52.8%).

Other frequently used technologies included video surveillance systems (*n* = 17, 32.1%) and milking robots or automated parlours (*n* = 16, 30.2%). Moderate adoption rates were observed for TMR pushing systems (*n* = 12, 22.6%), barn environmental monitoring systems (*n* = 11, 20.8%), and pedometer sensors (*n* = 10, 18.9%).

Less common technologies included NIR systems for ration analysis (*n* = 7, 13.2%), manure removal robots (*n* = 7, 13.2%), and integrated systems within the mixer wagon (*n* = 7, 13.2%). Minor adoption levels were recorded for ear tag sensors (*n* = 4, 7.5%), water consumption monitoring systems (*n* = 3, 5.7%), tail sensors (*n* = 2, 3.8%), body weight evaluation systems (*n* = 2, 3.8%), and only one farm (1.9%) each used BCS evaluation sensors or concentrate distribution systems for precision feeding.

### 3.3. Satisfaction with Current Technologies

**Figure 12:**
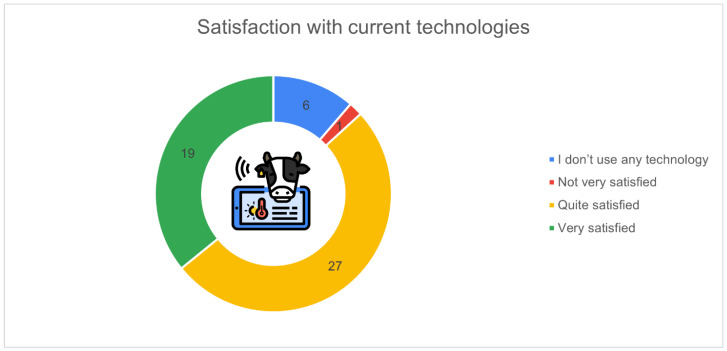
Satisfaction with current technologies (*n* = 53): quite satisfied (*n* = 27, 50.9%), very satisfied (*n* = 19, 35.8%), no technology use (*n* = 6, 11.3%), not very satisfied (*n* = 1, 1.9%).

Figure 12 summarises the level of satisfaction among respondents regarding the technologies currently used on their farms. Most participants reported being either quite satisfied (*n* = 27, 50.9%) or very satisfied (*n* = 19, 35.8%) with the systems in use. A smaller portion of respondents (*n* = 6, 11.3%) indicated that they do not use any technology, while only one farmer (1.9%) described themselves as not very satisfied.

The apparent discrepancy between Figure 11 and Figure 12 is due to differences in question structure. Figure 11 reports the selection of individual technologies among respondents who indicated usage of specific tools, whereas Figure 12 reflects the overall self-perception of technology use. Some respondents who selected individual technologies may still have considered their level of adoption as limited or insufficient, thus reporting that they do not use “technology” in a broader sense.

### 3.4. Main Limitations of Current Technologies

**Figure 13:**
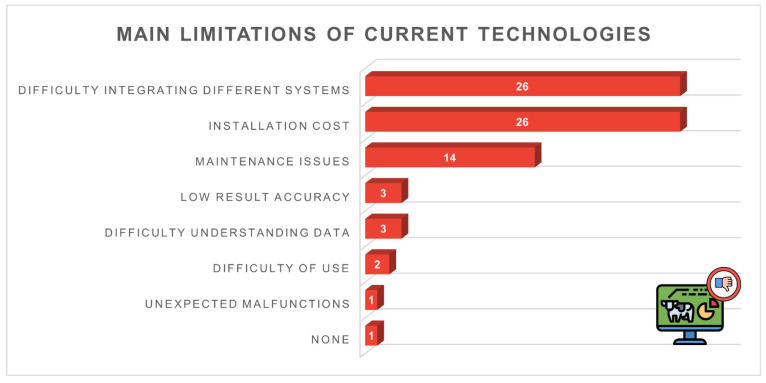
Main limitations of the technologies currently used (*n* = 53): system integration (*n* = 26, 49.1%), installation cost (*n* = 26, 49.1%), maintenance (*n* = 14, 26.4%), low accuracy (*n* = 3, 5.7%), data interpretation difficulty (*n* = 3, 5.7%).

Figure 13 summarises the main limitations of the technologies currently used on the farms. The two most frequently reported constraints were difficulty integrating different systems (*n* = 26, 49.1%) and installation cost (*n* = 26, 49.1%), both cited by nearly half of the respondents. Maintenance issues were mentioned by 14 participants (26.4%), while a smaller number of farmers indicated low result accuracy (*n* = 3, 5.7%) or difficulty in understanding data (*n* = 3, 5.7%).

### 3.5. Desired Monitoring Functions

**Figure 14:**
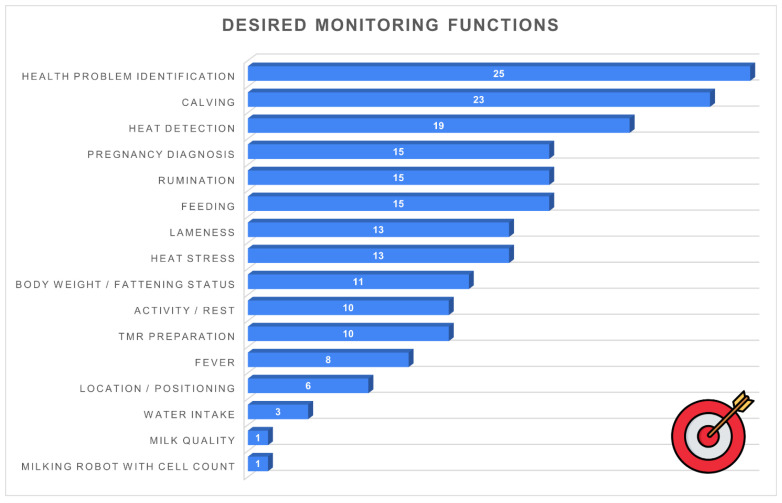
Desired monitoring functions for future PLF devices (*n* = 53): health problem detection (*n* = 25, 47.2%), calving detection (*n* = 23, 43.4%), heat detection (*n* = 19, 35.8%), pregnancy/rumination/feeding behaviour (*n* = 15, 28.3% each), lameness (*n* = 13, 24.5%), heat stress (*n* = 13, 24.5%), body weight/fattening status (*n* = 11, 20.8%), activity/rest (*n* = 10, 18.9%), TMR management (*n* = 10, 18.9%), fever (*n* = 8, 15.1%), positioning (*n* = 6, 11.3%), water intake (*n* = 3, 5.7%), milk quality (*n* = 1, 1.9%), robotic milking with SCC sensors (*n* = 1, 1.9%).

Figure 14 reports the main functions that farmers would like to see included in future monitoring devices. The most frequently mentioned needs were health problem identification (*n* = 25, 47.2%) and calving detection (*n* = 23, 43.4%), followed by heat detection (*n* = 19, 35.8%). Several respondents also expressed interest in monitoring pregnancy diagnosis, rumination, and feeding behaviour (each *n* = 15, 28.3%).

Other relevant areas of interest included lameness detection (*n* = 13, 24.5%), heat-stress monitoring (*n* = 13, 24.5%), and body weight or fattening status (*n* = 11, 20.8%). Functions related to activity and rest and TMR preparation were each selected by 10 respondents (18.9%).

Less common but still notable requests included fever detection (*n* = 8, 15.1%), location or positioning systems (*n* = 6, 11.3%), and water intake monitoring (*n* = 3, 5.7%). Only one respondent (1.9%) indicated interest in milk quality monitoring and milking robots with integrated cell count sensors.

### 3.6. Previous Collaborations in Research or Trials

**Figure 15:**
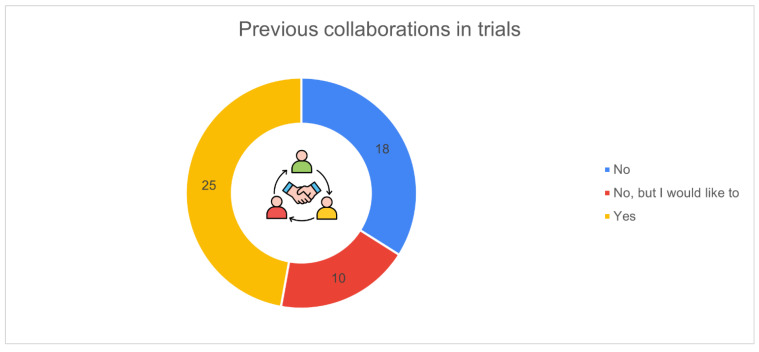
Previous participation in research or commercial trials (*n* = 53): yes (*n* = 25, 47.2%), no (*n* = 18, 34.0%), not yet but interested (*n* = 10, 18.9%).

Figure 15 shows the distribution of respondents according to their previous involvement in research or experimental collaborations with universities or private companies. More than one third of participants (*n* = 25, 47.2%) reported having already taken part in experimental or research activities, while 18 respondents (34.0%) indicated that they had never participated.

Interestingly, 10 farmers (18.9%) stated that they had not yet collaborated but would like to in the future, highlighting an openness to future engagement between the research community and the farming sector.

### 3.7. Relationship Between Herd Size and Investments in Technology

Among the 53 total questionnaires received, 37 respondents supplied quantifiable information on past investments, while 27 provided quantifiable data regarding planned investments. To evaluate the relationship between technological investments and herd size, farms were classified into two predefined categories based on the number of lactating cows: small farms ≤60 cows, and medium-large farms ≥61 cows. In the present dataset, 19 farms fell into the ≤60 lactating cows category, while 34 farms were classified in the ≥61 lactating cows category.

The distribution of technological investments made in the past five years differed between farms with ≤60 and ≥61 lactating cows. The median investment among smaller farms (≤60 cows) was €47,500, whereas larger farms (≥61 cows) reported a median of €200,000. The interquartile range (IQR) was markedly wider in larger herds (€37,500–€350,000) than in smaller ones (€5000–€176,250), indicating greater variability in past expenditure among larger operations. The Mann–Whitney U test indicated a statistically significant difference between the two groups (U = 91, z = −1.9056, *p* = 0.0284), suggesting that larger farms tended to invest more heavily in PLF technologies during the preceding five-year period. A similar pattern was observed for planned investments over the next five years. The median intended investment for smaller farms was €20,000, while larger farms reported a substantially higher median of €300,000, with future planned spending showing pronounced variability among larger herds, reaching values up to €2,000,000. The difference between the two herd-size groups was also statistically significant for planned investments (U = 36.5, z = −2.081, *p* = 0.0187).

### 3.8. Relationship Between Past and Future Investments

For the correlation analysis between past and planned investments, only respondents who provided quantifiable data for both variables were included. To assess whether previous spending patterns influenced future investment intentions, a bivariate correlation and a linear regression analysis were conducted. Past investments over the previous five years and planned investments for the upcoming five years were strongly and positively correlated (r = 0.9079, 95% CI: 0.8059–0.9575, *p* < 0.0001). This strong association indicates that farms which invested more in the recent past are also those planning higher expenditure in the near future. The covariance between the two variables was similarly high (1.11 × 10^11^), further supporting the consistency of this relationship.

A simple linear regression model was then applied to evaluate whether past investments statistically predicted future investment intentions. The model was highly significant (F = 117.22; *p* < 0.0001) and accounted for 82.4% of the variance in planned spending (R^2^ = 0.824).

The resulting regression equation was as follows:

Planned 5-year investment = 1437.56 + 1.72596 × Past 5-year investment

The slope coefficient was significant (β = 1.72596; *p* < 0.0001), indicating that for every euro invested in the previous five years, farmers intended to invest an additional 1.73 euros in the subsequent five-year period. The intercept was not significant (*p* = 0.978), suggesting no independent baseline effect beyond past expenditure.

### 3.9. Relationship Between Age Group and Investments in Technology

The distribution of past and planned investments across the five respondent age categories (<25, 26–35, 36–45, 46–55, >55 years) is summarised. Overall, both investment variables displayed substantial variability within and between age groups.

For past 5-year investments, the highest median values were observed in the 36–45 age group (€200,000), followed closely by respondents aged 46–55 (€175,000). Farmers under 25 reported a median investment of €42,500, whereas those aged 26–35 invested a median of €100,000. No valid responses were recorded in the >55 group. Interquartile ranges also varied widely, with the 75th percentile ranging from €162,000 (<25) to €375,000 (36–45).

For planned 5-year investments, a similar pattern of increasing investment capacity with age emerged. Respondents aged 36–45 again showed the highest median planned expenditure (€300,000), followed by the 26–35 group (€200,000). Farmers under 25 reported a median intended investment of €45,000, while the 46–55 group indicated a median of €100,000. As with past investments, no planned investment data were available for the >55 category. The upper quartile values ranged from €281,000 (<25) to €1,100,000 (36–45), indicating large differences in future spending intentions across age brackets (Table 1).

### 3.10. Investment Distribution by Geographical Macro-Areas

An additional analysis was conducted to assess whether past and planned investments in PLF technologies differed between farms located in the North of Italy and those located in Centre, South and Islands. Farms were grouped into two predefined macro-area categories: North and Centre, South, Islands. Based on this classification, 38 farms were located in the North, while 15 farms belonged to the Centre, South and Islands group.

Farms located in the Centre, South and Islands reported markedly higher median investments over the past five years compared with those in the North. The median investment was €200,000 for Centre-South-Islands farms, while northern farms reported a median of €80,000. The IQR showed substantial variability within both groups, but with upper quartiles reaching €600,000 in the Centre-South-Islands group compared with €200,000 in the North, suggesting that a subset of southern and island farms made particularly high investments. The Mann–Whitney U test confirmed a statistically significant difference between the two macro-areas (U = 181, z = 2.0517, *p* = 0.0402)

A similar pattern emerged for planned investments. Farms in the Centre, South and Islands reported a higher median planned investment (€500,000) compared with northern farms (€100,000). Variability remained substantial in both groups, with maximum declared future investments reaching €1,000,000 for the Centre-South-Islands and €2,000,000 for the North. Statistical testing showed a tendency toward significance: the Mann–Whitney U test yielded (U = 196, z = 1.7612, *p* = 0.0782). Although not fully significant at the 0.05 threshold, these values indicate a consistent trend toward higher future investment intentions among farms located in central and southern Italy.

### 3.11. Stratified Analysis of Technology Adoption

To further explore how structural and demographic factors influenced the adoption of PLF technologies, a stratified descriptive analysis was performed considering herd size, geographical macro-area, and education level. Farms with ≥61 lactating cows showed a higher frequency of adoption of advanced PLF technologies, particularly wearable sensors, automated milking systems, and environmental monitoring tools, compared to smaller farms (≤60 cows), which more frequently relied on basic mechanisation and standalone systems. A similar pattern was observed across geographical macro-areas. Farms located in the North reported a higher prevalence of integrated and automated technologies, whereas farms in the Centre, South, and Islands showed more heterogeneous adoption patterns, with a combination of basic and advanced systems. Education level also appeared to influence technology use. Respondents with a university degree reported more frequent adoption of multiple PLF tools and greater engagement with data-driven systems compared to those with lower educational levels, who more often reported the use of fewer or less integrated technologies. Although these patterns were not formally tested through multivariable models due to sample size limitations, they provide useful insights into how structural and human factors may influence digitalisation pathways in dairy farming.

## 4. Discussion

### 4.1. Adoption Trends and Structural Factors

The results of this study provide a broad and detailed overview of the current state of PLF adoption in Italian dairy farms and of the geographical factors that shape its development. The territorial origin of respondents offers an important interpretative lens: a substantial share of farms is in northern Italy, an area historically characterised by intensive dairy production, strong supply-chain integration and greater exposure to technological innovation. This pattern is consistent with the structural characteristics of the national dairy sector, where northern regions host a high density of medium to large scale farms and represent the core of Italian milk production, an aspect also reflected in previous surveys on PLF adoption in Italy [17,18]. However, the dataset also includes farms situated across central regions, southern areas and the islands, as well as in hilly and mountainous environments. This diversity indicates that digitalisation is not confined to highly intensive systems. The participation of farms located outside the traditional dairy strongholds suggests that PLF is increasingly perceived as a relevant tool for improving monitoring, efficiency and decision-making, even in areas where dairy farming may be more extensive, fragmented, or constrained by environmental and logistical challenges [19]. Comparable trends were observed by Abeni et al. [17], who conducted their survey exclusively in a single northern province (Cremona, Lombardy), one of the most intensive dairy districts in the country, thus offering a geographically concentrated picture of PLF adoption that contrasts with the broader national distribution captured in the present study. Similarly, Bianchi et al. [18] reported findings largely based on Lombardy dairy farms (approximately 79% of their sample), reflecting strong technological penetration in northern intensive systems, although their dataset included far fewer respondents from central and southern regions than the present work.

When the territorial data are considered in a broader geographical perspective, a clear asymmetry in adoption intensity emerges, with northern Italy representing the primary hub of PLF uptake, while central, southern and island regions contribute a smaller share of users. This pattern reflects the structural characteristics of the Italian dairy sector but also indicates that PLF adoption is expanding beyond traditional intensive regions, although at different speeds depending on local production contexts. The type of terrain in which farms operate is another relevant aspect. The predominance of farms located in plains was expected, as these areas host the largest concentration of intensive dairy enterprises. Nonetheless, the meaningful presence of farms in hilly and mountain territories indicates that digitalisation is gaining relevance even in environments where daily management is more challenging due to structural or logistical constraints. In such contexts, technology may play an even more strategic role by compensating for limited labour availability or supporting animal monitoring in less standardised environments [20]. Neither Abeni et al. [17] nor Bianchi et al. [18] incorporated terrain-based stratification, making this dimension a novel contextual contribution of the present study.

Farm size diversity within the sample further confirms that digitalisation is not a prerogative of large farms. Similar proportions of farms between 10 and 30 hectares, 30–50 hectares, and over 100 hectares indicate that interest in PLF spans across different farm typologies. This heterogeneity is also reflected in herd size, with small herds coexisting with farms managing more than 200 lactating cows. Nevertheless, statistical analyses showed that future investments tend to be influenced by herd size, an expected result considering that larger herds carry greater management risks and benefit more from automated monitoring tools. This tendency aligns with Abeni et al. [17], who also reported greater interest in PLF among larger dairy barns. These results are also consistent with international evidence. In Irish pasture-based dairy farms, herd size was also identified as one of the strongest predictors of PLF adoption intensity, suggesting that this relationship holds across structurally different production systems [21].

From a structural standpoint, the predominance of free-stall housing reflects an already advanced phase of modernisation. This is particularly important because housing type directly affects the feasibility and performance of many PLF technologies, especially wearable sensors and distributed environmental monitoring systems. The marginal presence of tie-stall barns confirms an ongoing shift towards more flexible, technology-compatible systems. Similarly, the strong predominance of Holstein cows contributes to a certain standardisation in technological needs, facilitating implementation of systems calibrated to a well-defined biological model [9]. Nonetheless, the substantial presence of Simmental, Brown Swiss, and crossbred herds demonstrates that farms with different genetic profiles also perceive PLF tools as valuable. Unlike herd size, breed distribution was not a focal point in Abeni et al. [17], and Bianchi et al. [18] did not provide comparative analyses by breed; therefore, the broader inclusion of minor breeds in the present study adds contextual diversity not detailed in earlier Italian PLF surveys.

The sociodemographic characteristics of respondents add another important interpretative layer. The dominance of farm owners or managers among participants implies that the responses mainly reflect the perspectives of individuals responsible for investment decisions. The strong representation of farmers aged 26–45 and the high proportion of respondents with medium-to-high educational levels point to a group actively engaged in technical and managerial renewal. Furthermore, the fact that two-thirds of respondents have an educational background related to agriculture or livestock sciences helps explain the observed willingness to adopt digital technologies, as higher competencies facilitate data interpretation and recognition of the value of PLF tools. Bianchi et al. [18] similarly identified younger farmers as key drivers of technological uptake, although they did not examine education level in detail, making the present analysis complementary to their demographic insight. Similar associations have been observed in Irish dairy farms; younger farmers, higher agricultural education and a greater reliance on hired labour significantly increased the likelihood of adopting multiple PLF technologies [21].

### 4.2. Technology Use and Farmer Needs

The section on current technology use confirms that digitalisation is already well established among the surveyed farms. The widespread use of management software, automated TMR preparation systems and heat-stress mitigation technologies indicates that farmers prioritise tools with direct impact on management efficiency [22]. More than half of respondents also use collar sensors, reflecting the growing importance of behavioural monitoring [23]. At the same time, the limited adoption of advanced technologies such as NIR systems or fully integrated mixer-wagon tools suggests that more sophisticated forms of digitalisation are progressing gradually and unevenly [24]. Overall, these trends are broadly consistent with those reported by Abeni et al. [17] and Bianchi et al. [18], although the present study covers a wider range of technologies, including environmental sensors, precision concentrate feed tools, and weight-evaluation systems, that were not a central focus of previous Italian questionnaires, thereby providing a more comprehensive overview of PLF implementation.

Satisfaction with current technologies was generally high, demonstrating their perceived usefulness in daily management. However, identified limitations reveal a gap between the theoretical potential of PLF and real on-farm conditions. Nearly half of respondents cited lack of integration between systems as a major barrier, an issue widely recognised at international level and strongly limiting the full value of digital data [1].

The difficulty in integrating different PLF systems likely reflects several underlying technical challenges, including vendor-specific data ecosystems that limit interoperability (vendor lock-in), lack of standardised data formats, restricted access to application programming interfaces (APIs), and the presence of fragmented data streams (“data silos”). These factors increase the complexity of data interpretation and reduce the effective usability of digital tools.

From a technological perspective, these interoperability challenges could be addressed through the adoption of middleware architectures and open communication standards. Protocols such as MQTT (Message Queuing Telemetry Transport) or CoAP (Constrained Application Protocol), widely used in Internet of Things (IoT) systems, enable efficient and standardised data exchange between heterogeneous devices. The implementation of such solutions could facilitate the integration of multiple PLF systems into unified platforms, reducing data fragmentation and improving the scalability and usability of digital infrastructures on farms.

Installation cost was also a critical concern, confirming that adoption is strongly shaped by farm economics, which in turn depend on factors such as farm size, production model and financial capacity. Both aspects were also acknowledged in Abeni et al. [17] and Bianchi et al. [18], and the present study extends these observations by quantifying the proportion of farmers who identify each barrier as critical, thereby clarifying their relative importance within the surveyed population. Comparable motivations and barriers have been reported in pasture-based production systems. Irish farmers highlighted labour-saving benefits and improvements in milking efficiency as key drivers of adoption, while cost, data interpretation challenges and concerns over system accuracy limited wider uptake [21]. The convergence of these findings suggests that, despite structural differences between indoor and grazing-based systems, farmers share similar expectations for intuitive, economically sustainable and decision-supportive technologies.

The functional priorities expressed by farmers provide important insights into where PLF development should focus on the coming years. The strong demand for tools enabling early detection of diseases, calving, heat events and lameness aligns closely with the most critical aspects of herd management and confirms widespread awareness of the preventive value of PLF systems. Interest in functions related to rumination, activity, and heat-stress monitoring further reflects growing attention to animal welfare, now considered an integral component of farm sustainability in Italy. These priorities are coherent with those reported by Bianchi et al. [18], who similarly identified health, reproduction and welfare monitoring as key areas of technological interest.

The priorities expressed by farmers align closely with current developments in artificial intelligence applied to livestock farming. Some applications, such as heat detection and rumination monitoring based on sensor data, are already supported by commercially available systems and can be considered near-deployable technologies. In contrast, more complex applications, such as automated lameness detection or early disease prediction using multimodal datasets (e.g., behaviour, production and environmental data), remain largely at the research or pilot stage and require further validation under commercial conditions. This distinction highlights the need to align technological innovation with both data availability and practical usability on farms.

Willingness to collaborate with research institutions or private companies offers another indicator of maturity within the sector. Almost half of the respondents had previously participated in trials, suggesting that a significant portion of Italian dairy farmers view innovation not as an external imposition but as a concrete opportunity. The additional group expressing interest in future participation further reinforces this potential, indicating an environment favourable to co-development of technologies. Abeni et al. [17] also reported a general openness toward innovation among farmers, although collaboration with research partners was not a topic explored in their study.

### 4.3. Investment Behaviour

The section on investments carries managerial and economic significance. This economic dimension represents a key advancement over previous Italian PLF surveys, as neither Abeni et al. [17] nor Bianchi et al. [18] quantified PLF-related investments in monetary terms or modelled the relationship between past and future investment intensity. The association between herd size and both past and future investments reflects a well-documented trend: larger farms invest more, both out of necessity and because they have greater financial capacity. However, the correlation and regression analyses linking past and future investments reveal an even more relevant insight: past investment intensity is a strong predictor of future investment. The relationship was not only linear but also economically substantial, as the model showed that for every euro invested in the previous five years, farmers planned to invest an additional 1.73 euros in the next five-year period. This pattern indicates that digitalisation in dairy farming tends to follow cumulative trajectories: farms that have already invested are the ones most likely to continue investing, progressively increasing their technological advancement. Such dynamics risk widening the digital divide between highly innovative farms and those less inclined to adopt new technologies, potentially segmenting the sector into early adopters and more static enterprises. This cumulative investment pattern parallels the “adoption intensity” framework described by Palma-Molina et al. [21], where farms already using several technologies tended to continue expanding their digital portfolio. Although the present study did not classify respondents into adoption clusters, the strong correlation between past and future investments suggests that Italian farms may follow similar stepwise digitalisation directions.

Age group analysis adds another layer, showing that the greatest investment potential is found among farmers aged 36–45, who appear to represent a balance between experience, economic stability and openness to innovation. Younger farmers reported lower investment levels, likely due to recent entry into managerial roles or limited financial resources, while older farmers showed lower engagement in future investments, possibly reflecting shorter planning horizons. Bianchi et al. [18] also found that younger and mid-career farmers were more inclined to adopt PLF technologies, although they did not evaluate investment capacity directly; the present work therefore provides additional context to this demographic pattern.

An additional insight from the analysis concerns the influence of geographical macro-areas on technological investment behaviour. Contrary to expectations, given that northern dairy regions typically have larger herds, higher production intensity and stronger economic structures, the results showed an opposite pattern: farms in the Centre, South and Islands reported higher median investments, both in the previous five years and in their planned expenditure. Several factors may account for this trend. Farms in these regions may adopt PLF technologies as a strategic means to compensate for structural or logistical constraints, labour shortages or more variable production contexts, using innovation to improve efficiency and competitiveness. Another plausible explanation is the impact of targeted public funding. Over recent years, national and regional schemes (e.g., Piano di Sviluppo Rurale, Fondo Innovazione, Nuova Sabatini, Transizione 4.0, and incentives dedicated to southern regions) have channelled substantial resources into supporting technological upgrades in agriculture. Farms in central and southern areas, often prioritised for development, may therefore have benefited disproportionately, enabling higher investment levels irrespective of herd size. Although differences in future planned investments between macro-areas did not reach full statistical significance, the trend remained evident. This geographical investment pattern had not been previously examined in Italian PLF surveys, which were geographically limited or regionally unbalanced, and therefore represents a distinct contribution of the present study. Regional variation has also been documented in other national contexts. In Ireland, Palma-Molina et al. [21] reported marked geographical differences in the probability of adopting PLF tools, supporting the idea that local structural and socio-economic conditions strongly influence digitalisation patterns. This alignment suggests that territorial heterogeneity is a broader feature of PLF adoption rather than one confined to Italy.

Overall, the findings suggest that digitalisation in Italian dairy farming is an ongoing and tangible process, but one marked by substantial internal heterogeneity. Better resourced farms led by younger, well-trained individuals appear to be driving PLF adoption, while structural and operational challenges still hinder more uniform diffusion. The needs expressed by farmers clearly indicate the direction in which future technologies should evolve: systems that are more integrated, more reliable, easier to use, and capable of delivering information directly relevant to daily herd management.

### 4.4. Limitations and Future Directions

This study has some limitations that must be considered when interpreting the results. The sample size (*n* = 53), although adequate for descriptive purposes, limits the generalisability of the findings to the wider Italian dairy sector. In addition, the sampling strategy was based on voluntary participation and dissemination through social media channels and specialised agricultural media, which may have introduced elements of convenience sampling bias. This approach may have favoured the inclusion of farmers who are more digitally engaged, innovation-oriented, or already interested in Precision Livestock Farming technologies, potentially leading to an overestimation of adoption levels and investment intentions. Although the sample included farms with a range of herd sizes and geographical areas, it was not designed to be statistically representative of the national dairy population, and therefore caution is required when generalising the results. The exclusive use of an online questionnaire may have further limited participation from less digitally connected farmers. Furthermore, some subgroups, such as respondents over the age of 55, were underrepresented in the investment analyses, and reliance on self-reported data may have introduced recall or subjective bias. Future research should aim to strengthen external validity by adopting more structured sampling strategies and aligning sample characteristics, such as herd size and regional distribution, with national agricultural statistic.

Despite these limitations, the study provides an up-to-date and meaningful picture of the adoption of PLF in Italian dairy farms and offers several indications for future work. Farmers’ responses highlight the need for more intuitive, better integrated technologies that can reduce the cognitive load associated with fragmented dashboards and heterogeneous alert systems. Future research should therefore focus on how management software and sensor platforms are used in practice, identifying usability limitations and opportunities for the development of unified interfaces that simplify data interpretation and support decision-making. Improving data interoperability, including shared architectures, standardised communication protocols and the efficient integration of heterogeneous data flows, emerges as a priority for translating the potential of PLF into effective application on farms.

Further studies should also explore the socio-economic and territorial factors that determine technology adoption through longitudinal and region-specific analyses. Mixed methodological approaches combining surveys, interviews and observational research will be essential to capture farmers’ motivations, expectations and real-time interactions with digital tools.

## 5. Conclusions

This study confirms that precision farming is already widely adopted by Italian dairy farms, particularly through management software, automated feeding systems, heat stress mitigation technologies, and wearable sensors. The high levels of satisfaction reported indicate that these tools are perceived as effective aids to day-to-day management and not as experimental solutions. However, the persistent challenges of high installation costs and poor interoperability between systems remain important obstacles to fully leveraging the potential of digital technologies, often limiting the integration of data into coherent and user-friendly decision support frameworks. Analysis of investment patterns highlights the presence of cumulative digitalisation pathways within the sector. Larger farms invest significantly in PLF technologies, and past expenditure is a strong indicator of future investment intentions.

Farmers’ priorities for future monitoring devices focus primarily on early diagnosis of health problems, calving, reproductive events and lameness, followed by monitoring of feeding and rumination, reflecting a strong orientation towards functions of immediate relevance to animal productivity and welfare. The willingness of many respondents to engage in research and commercial collaborations further indicates favourable conditions for participatory innovation. Overall, Italian dairy farming appears to be transitioning from adoption to consolidation of PLF technologies, highlighting the need for more integrated, accessible and farmer-centred solutions to support sustainable and inclusive digital development in diverse agricultural contexts.

## Figures and Tables

**Table 1:** Past and planned investment distribution by age class. The table reports past and planned 5-year investments across all age categories used in the survey (<25, 26–35, 36–45, 46–55 and >55 years). For each age class, the table includes the median, 25th percentile and 75th percentile for both past and planned investments.

		Age				
		<25	26–35	36–45	46–55	>55
**Past 5 years investment**	Median	42,500	100,000	200,000	175,000	/
	Quantiles25	23,750	25,000	21,250	112,500	/
	Quantiles75	162,000	300,000	375,000	275,000	/
**Planned 5 year investment**	Median	45,000	200,000	300,000	100,000	/
	Quantiles25	10,000	30,000	110,000	0	/
	Quantiles75	281,000	500,000	1,100,000	300,000	/

## Data Availability

The datasets presented in this study are not publicly available due to privacy considerations but are available from the corresponding author upon reasonable request.

## Supplementary Materials

The following supporting information can be downloaded at: https://www.mdpi.com/article/10.3390/ani16081170/s1. Questionnaire S1: Full questionnaire administered to respondents.

## Abbreviations

The following abbreviations are used in this manuscript:

AI	Artificial Intelligence
BCS	Body Condition Score
CI	Confidence Interval
GDPR	General Data Protection Regulation
IQR	Interquartile Range
NIRS	Near-Infrared Spectroscopy
PLF	Precision Livestock Farming
TMR	Total Mixed Ration
