# Supplementary material for: Smart Enough? What Italian Farmers Reveal About Dairy Cow Technologies: A Survey Study
Source: Animals (Basel). 2026 Apr 11;16(8):1170. doi: 10.3390/ani16081170 (PMC13113817; doi:10.3390/ani16081170)
Supplement: Supplementary file 1 [file animals-16-01170-s001.zip › animals-4190695-supplementary.pdf]

SUPPLEMENTARY MATERIAL

SMART DEVICES FOR DAIRY COWS - FARMER QUESTIONNAIRE

Dear farmer,
this questionnaire has been designed to investigate the adoption, limitations, and expectations regarding smart, multi-sensor technologies for monitoring dairy cows. Completing it takes approximately 10 minutes. Responses are anonymous, and personal data will be used only if voluntarily provided.

Questions marked with an asterisk (*) are mandatory.

SECTION 1 - GENERAL INFORMATION

1. Province where the farm is located *

(Open answer)

2. Geographical area where the animals are kept *

(Select one option only)
- Plain
- Hill
- Mountain

3. Farm area (hectares) *

(Select one option only)
- <10
- 10-30
- 30-50
- 50-100
- >100

4. Number of lactating cows *

(Select one option only)
- <30
- 31-60
- 61-100
- 101-200
- >200

5. Housing system *

(Select one option only)
- Pasture-based
- Tie-stall
- Free-stall
- Mixed

6. Breeds present on the farm *

(Select all that apply)
- Holstein
- Simmental
- Brown Swiss
- Jersey
- Crossbreeds
- Other: ________

7. What is your role within the farm? *

(Select one option only)
- Owner
- Herd manager
- Employee

8. How old are you? *

(Select one option only)
- <25
- 26-35
- 36-45
- 46-55
- >55

9. What is your highest level of education? *

(Select one option only)
- Middle school certificate
- High school diploma
- University degree
- Other: ________

10. Is your educational background related to your current work activity? *

(Select one option only)
- Yes
- No

SECTION 2 - TECHNOLOGIES CURRENTLY USED ON THE FARM

11. Which technologies do you currently use? *

(Select all that apply)

- Management software
- Milking robot / automated milking parlor
- Collar sensors
- Pedometer sensors
- Ear-tag sensors
- Tail sensors
- Body-weight evaluation systems
- BCS (body condition score) evaluation sensors
- Barn environmental condition evaluation systems
- Heat-stress mitigation systems

• TMR pushing systems
• TMR preparation systems (wagon/robot)
• Integrated systems in the mixer wagon
• Ration composition evaluation systems (NIR)
• Water consumption evaluation systems
• Manure removal robot
• Video surveillance systems
• Concentrate distribution system for precision feeding
• No technology in use
• Other: ________

12. Overall, how satisfied are you with the technologies you use? *

(Select one option only)

• Very satisfied
• Quite satisfied
• Not very satisfied
• Not at all satisfied
• I don’t use any technology

13. What are the main limitations of the technologies you use? *

(Select up to two)

• Installation cost
• Maintenance issues
• Difficulty of use
• Difficulty understanding data
• Low result accuracy
• Difficulty integrating different systems
• Unexpected malfunctions
• None
• Other: ________

14. How much have you invested in technologies over the past 5 years?

(Open answer - approximate amount in euros)

15. How much would you be willing to invest in the next 5 years?

(Open answer - approximate amount in euros)

SECTION 3 - NEEDS AND EXPECTATIONS

16. If you could invest in a new device, what should it monitor? (max 3 answers) *

(Select up to three)

• Feeding
• TMR preparation
• Rumination
• Activity / rest
• Health problem identification
• Heat detection

• Pregnancy diagnosis
• Fever
• Heat stress
• Lameness
• Water intake
• Location / positioning
• Body weight / fattening status
• Calving
• Milk quality
• Milking robot with cell count
• Other: ________

FINAL SECTION - COLLABORATION AND CONTACT

17. Have you ever taken part in surveys or studies carried out by universities or private companies? *

(Select one option only)

• Yes
• No
• No, but I would like to

18. Farm name (optional)

(Open answer)

19. Email and/or phone contact (optional - for updates about the study)

(Open answer)

GDPR Notice

In accordance with Article 13 of Regulation (EU) 2016/679 (GDPR), we inform you that any personal data collected through this questionnaire will be processed exclusively for research purposes and for the development of wearable devices for animals.
Data will be:
• processed lawfully, fairly and transparently;
• used only for the purposes explicitly stated in this survey;
• stored for the time strictly necessary to achieve those purposes;
• protected by appropriate technical and organisational measures to ensure confidentiality and security.

Providing contact details is optional; without them, updates on the study may not be sent.
